# HIF‐1α ameliorates tubular injury in diabetic nephropathy via HO‐1–mediated control of mitochondrial dynamics

**DOI:** 10.1111/cpr.12909

**Published:** 2020-09-25

**Authors:** Na Jiang, Hao Zhao, Yachun Han, Li Li, Shan Xiong, Lingfeng Zeng, Ying Xiao, Ling Wei, Xiaofen Xiong, Peng Gao, Ming Yang, Yu Liu, Lin Sun

**Affiliations:** ^1^ Department of Nephrology The Second Xiangya Hospital of Central South University Changsha China; ^2^ Hunan Key Laboratory of Kidney Disease and Blood Purification Changsha China

## Abstract

**Objectives:**

In diabetic nephropathy (DN), hypoxia‐inducible factor‐1α (HIF‐1α) activation in tubular cells plays an important protective role against kidney injury. The effects may occur via the target genes of HIF‐1α, such as haem oxygenase‐1 (HO‐1), but the exact mechanisms are incompletely understood.

**Materials and methods:**

Mice with proximal tubule‐specific knockout of HIF‐1α (PT‐HIF‐1α^−/−^ mice) were generated, and diabetes was induced in these mice by streptozotocin (STZ) injection. In addition, to mimic a hypoxic state, cobaltous chloride (CoCl_2_) was applied to HK‐2 cells.

**Results:**

Our study first verified that conditional knockout of HIF‐1α worsened tubular injury in DN; additionally, aggravated kidney dysfunction, renal histopathological alterations, mitochondrial fragmentation, ROS accumulation and apoptosis were observed in diabetic PT‐HIF‐1α^−/−^ mice. In vitro study showed that compared to control group, HK‐2 cells cultured under hypoxic ambiance displayed increased mitochondrial fragmentation, ROS production, mitochondrial membrane potential loss and apoptosis. These increases were reversed by overexpression of HIF‐1α or treatment with a HO‐1 agonist. Importantly, cotreatment with a HIF‐1α inhibitor and a HO‐1 agonist rescued the HK‐2 cells from the negative impacts of the HIF‐1α inhibitor.

**Conclusions:**

These data revealed that HIF‐1α exerted a protective effect against tubular injury in DN, which could be mediated via modulation of mitochondrial dynamics through HO‐1 upregulation.

## Graphical abstract

HIF‐1α is a transcription factor that regulates HO‐1 gene expression, and HO‐1 facilitates mitochondrial fusion (Mfn1 and Mfn2) and inhibits mitochondrial fission (Drp1 and Fis1), which maintains mitochondrial homeostasis. Under diabetic conditions, kidney tissues are in hypoxia, which induces the expression of HIF‐1α through an impaired compensatory response. However, tubular‐specific deletion of HIF‐1α in the kidneys of diabetic mice exaggerated the mitochondrial dysfunction and ROS accumulation and then caused tubular damage, thereby leading to diabetic kidney injury.

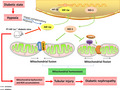

## 1 INTRODUCTION

There are multiple factors participate in diabetic nephropathy (DN) progression, such as oxygen metabolic disorder and glycometabolism disturbance. Among these factors, oxygen metabolic disorder is believed to be prominent in renal injury in DN, resulting in hypoxia, advanced glycation, oxidative stress and other deleterious effects.^1^, ^2^, ^3^ Furthermore, studies have implied that hypoxia is an upstream mediator of these phenomena that lead to DN, which frequently occurs in diabetic individuals.^1^, ^4^, ^5^ Nevertheless, the mechanisms underlying hypoxia‐induced kidney damage in DN are still unclear.

It is known that hypoxia‐inducible factor‐1 (HIF‐1) is a critical molecule for mitigating hypoxia‐induced damage and exists as a heterodimer comprising two subunits: a variable α‐subunit and a constitutively expressed β‐subunit. Moreover, the α‐subunit is typically rapidly degraded by the proteasome in normoxia and is stabilized in hypoxia.^6^, ^7^ HIF‐1 can perform its transcriptional function only subunits are complexed.^6^, ^7^, ^8^ Previous studies have shown that an oxygen deficit is present in DN and that enhancing HIF‐1 signalling ameliorates the progression of DN.^3^, ^9^, ^10^, ^11^ Although HIF‐1α likely plays a renoprotective role, its precise mechanisms in DN have not yet been fully elucidated.

Unsurprisingly, HIF‐1α has a connection with mitochondria because both are related to oxygen metabolism. It has been reported that HIF‐1α can stimulate the expression of genes encoding proteins involved in the mitochondrial tricarboxylic acid (TCA) cycle^12^ and autophagy^13^ regulation to improve mitochondrial morphology and function.^14^, ^15^ Previous studies by our laboratory showed the importance and characteristics of mitochondrial dynamics in DN,^16^, ^17^ and our results are consistent with those from other studies.^16^, ^18^, ^19^ More intriguingly, haem oxygenase‐1 (HO‐1), a target gene of HIF‐1α,^20^, ^21^, ^22^ has been reported to restrain hypoxia‐induced mitochondrial fission.^23^ Collectively, these findings highlight the possibility that HIF‐1α impacts mitochondrial function and morphology in kidneys of DN. However, the exact molecular mechanism by which HIF‐1α protects against mitochondrial dysfunction in tubular cells in the setting of DN is unknown.

In the present study, mice with proximal tubular cell‐specific genetic ablation of HIF‐1α were generated, and mice were induced to diabetes by streptozotocin (STZ) injection. In addition, a hypoxic cell model was established by treatment with cobaltous chloride (CoCl_2_), and these cells were then cultured and subjected to various treatments to investigate related mechanisms, aiming to exploring new therapeutic targets for DN.

## 2 MATERIALS AND METHODS

### 2.1 Generation of mice with conditional knockout of HIF‐1α

HIF‐1α‐floxed mice were purchased from the Jackson Laboratory. PEPCK‐Cre mice were kindly provided by Dr Tang (The Second Xiangya Hospital, China). Mice with targeted knockout of HIF‐1α in proximal tubular cells (PT‐HIF‐1α^−/−^ mice) were generated by the Cre‐LoxP recombination strategy. Cre‐negative littermate wild‐type mice (fl/fl mice) were used as controls. To genotype the mice, genomic DNA was extracted from tail tissues and detected as described previously.^24^ The sequences of the primers used were as follows: HIF‐1α forward primer (5′‐3′), TGCATGTGTATGGGTGTTTTG; HIF‐1α reverse primer (5′‐3′), GAAAACTGTCTGTAACTTCATTTCC; Cre forward primer (5′‐3′), ACCTGAAGATGTTCGCGATTATCT; Cre reverse primer (5′‐3′), ACCGTCAGTACGTGAGATATCTT.

### 2.2 Design in the animal experiment

Nine‐week‐old male PT‐HIF‐1α^−/−^ mice and littermate fl/fl mice were randomly divided into four groups: fl/fl mice, PT‐HIF‐1α^−/−^ mice, diabetic fl/fl mice and diabetic PT‐HIF‐1α^−/−^ mice. These mice were fasted overnight and then intraperitoneally injected with STZ (50 mg/kg body wt; Sigma‐Aldrich) consecutively for 5 days as previously described.^25^ Three days after the STZ injection, mice with blood glucose levels of >16.7 mmol/L were selected as diabetic mice for the experiment, and the mice were euthanized after 12 weeks. All mice were monitored by the Animal Care and Use Committee of Central South University (China), and all experiments were performed in precise accordance with the established regulations.

### 2.3 Assessment of metabolic and physiological parameters

The body weight and blood glucose level were measured biweekly, and blood and urine were collected before euthanasia. The blood glucose level and blood samples were assessed as described previously.^17^ Urine N‐acetyl‐β‐d glucosaminidase (NAG) was assessed using an automated colorimetric method (Pacific). Urinary creatinine and albumin were measured with a creatinine assay kit and an Albuwell M kit (Exocell).^24^

### 2.4 Histopathological analysis of kidney

Kidney tissue was excised, cut, fixed with paraformaldehyde and embedded in paraffin. Then, these kidney tissue blocks were sliced into four micrometre thick sections. To evaluate the kidney tissue damage, the sections were stained with haematoxylin‐eosin (HE) and periodic acid‐Schiff (PAS),^26^ and these changes were scored with a semiquantitative scoring system (0‐3).^27^

### 2.5 Immunohistochemical (IHC) staining

For IHC studies, paraffin‐embedded kidney sections were deparaffinized and hydrated using slide warmers and alcohol. After antigen retrieval, the sections were permeabilized with 3% H_2_O_2_ and blocked with 5% bovine serum albumin (BSA). Then, the sections were individually incubated with the following antibodies at appropriate concentrations: antibody against HO‐1 (ab13248) from Abcam and antibodies against Mfn1 (13798‐1‐AP), Mfn2 (12186‐1‐AP), Drp1 (12957‐1‐AP) and Fis1 (10956‐1‐AP) from Proteintech. Then, the sections were incubated with secondary antibodies and reacted with diaminobenzidine (DAB) in accordance with the manufacturer's instructions.^24^

### 2.6 Immunofluorescence (IF) staining

The above‐described paraffin‐embedded kidney sections were also used for IF staining according to previously published procedures.^26^ Briefly, the sections were successively labelled with an anti‐HIF‐1α primary antibody (ab179483) and the corresponding secondary antibody.

### 2.7 Establishment of a hypoxic cell model and treatment of cells

A human proximal tubular cell line (HK‐2) was acquired from ATCC, and cells were cultured in medium as described previously.^26^ To establish the hypoxic cell model, the cells were treated with 300 mmol/L CoCl_2_ for 24 hours to^28^ simulate hypoxia. In this model, cells were cultured with the HIF‐1α inhibitor KC7F2 (40 µmol/L) (APExBIO) for 24 hours to interfere with HIF‐1α expression. For overexpression of HIF‐1α, cells were transfected with a pHIF‐1α (401Δ603) plasmid (kindly provided by Dr Nicolas (Hospital Saint Louis, Paris, France)) using Lipofectamine 2000 according to the kit instructions.^24^ In addition, HK‐2 cells were treated with the HO‐1 agonist hemin (20 µmol/L) (Sigma) for 24 hours.

### 2.8 Examination of mitochondrial morphological changes by electron microscopy (EM) and fluorescence staining

Mitochondria in renal tubules were observed by EM as previously described.^17^ HK‐2 cells were stained with MitoTracker Red (Life Technologies) as previously described.^17^ HK‐2 cells stained with MitoTracker Red were labelled with anti‐Drp1 (ab184247) and anti‐HO‐1 (ab13248) antibodies for colocalization assays.

### 2.9 Analysis of apoptosis and reactive oxygen species (ROS) production

Paraffin‐embedded sections were subjected to terminal deoxynucleotidyl transferase dUTP nick end‐labelling (TUNEL) following the manufacturer's instructions.^29^ A dihydroethidium (DHE) (Invitrogen) probe and MitoSox Red (Invitrogen) were used to evaluate intracellular ROS accumulation in renal tubular tissues and HK‐2 cells, respectively.

### 2.10 Western blot analysis

Renal tissue and HK‐2 cells were collected, and total protein was extracted. In addition, mitochondrial and cytoplasmic proteins from HK‐2 cells were isolated using a Cell Mitochondria Isolation Kit according to the manufacturer's instructions.^26^ Primary proximal tubular epithelial cells were isolated from mice (Method S1), and total protein was extracted. A total of 30 µg of protein were loaded onto an 8%‐12% gel and separated via sodium dodecyl sulphate (SDS)‐polyacrylamide gel electrophoresis (PAGE). Then, proteins were transferred to a membrane and incubated with the following primary antibodies at the appropriate concentrations: antibodies against HIF‐1α (ab2185) and HO‐1 (ab13248); antibodies against Mfn1 (13798‐1‐AP), Mfn2 (12186‐1‐AP), Drp1 (12957‐1‐AP), Fis1 (10956‐1‐AP), Caspase‐9 (66169‐1‐Ig), Bax (60267‐1‐Ig), Cytochronmed C (Cytoc) (66264‐1‐Ig), COXIV (11242‐1‐AP) and β‐actin (20536‐1‐AP) from Proteintech; antibodies against p‐Drp1 (3455S) and cleaved Caspase‐3 (9661S) from Cell Signaling Technology.

### 2.11 Real‐time PCR

Total RNA from HK‐2 cells in different intervention groups was isolated using TRIzol reagent. A Prime Script Reagent kit (Takara) was used according to the manufacturer's instructions to synthesize cDNA from the extracted RNA. Then, according to the instructions, SYBR GreenER qPCR SuperMix (Thermo) was used to reverse transcribe the cDNA in a 7300 Real‐Time PCR System (Applied Biosystems).^24^ The following primers were used for amplification: HO‐1 forward primer (5′‐3′), GCCATGAACTTTGTCCGGTG; HO‐1 reverse primer (5′‐3′), TTTCGTTGGGGAAGATGCCA; β‐actin forward primer (5′‐3′), CATGTACGTTGCTATCCAGGC；β‐actin reverse primer (5′‐3′), CTCCTTAATGTCACGCACGAT.

### 2.12 Mitochondrial membrane potential (ΔΨm) assay

The mitochondrial membrane potential was evaluated with a specific dye. In brief, HK‐2 cells were stained with tetramethylrhodamine (TMRE, Molecular Probes) at a final concentration of 1 µmol/L for 30 minutes and were then visualized with confocal microscopy.

### 2.13 Statistical analyses

The statistical significance of the difference between two groups was evaluated by an unpaired *t* test in animal and cell studies, and the significance of the difference was denoted by the *P* value (*P* < .05, *P* < .005, *P* < .001). All statistical analyses were implemented in Graph Pad Prism 8.

## 3 RESULTS

### 3.1 Targeted deletion of HIF‐1α in proximal tubular cells aggravates kidney injury in diabetic mice

**Figure 1:**
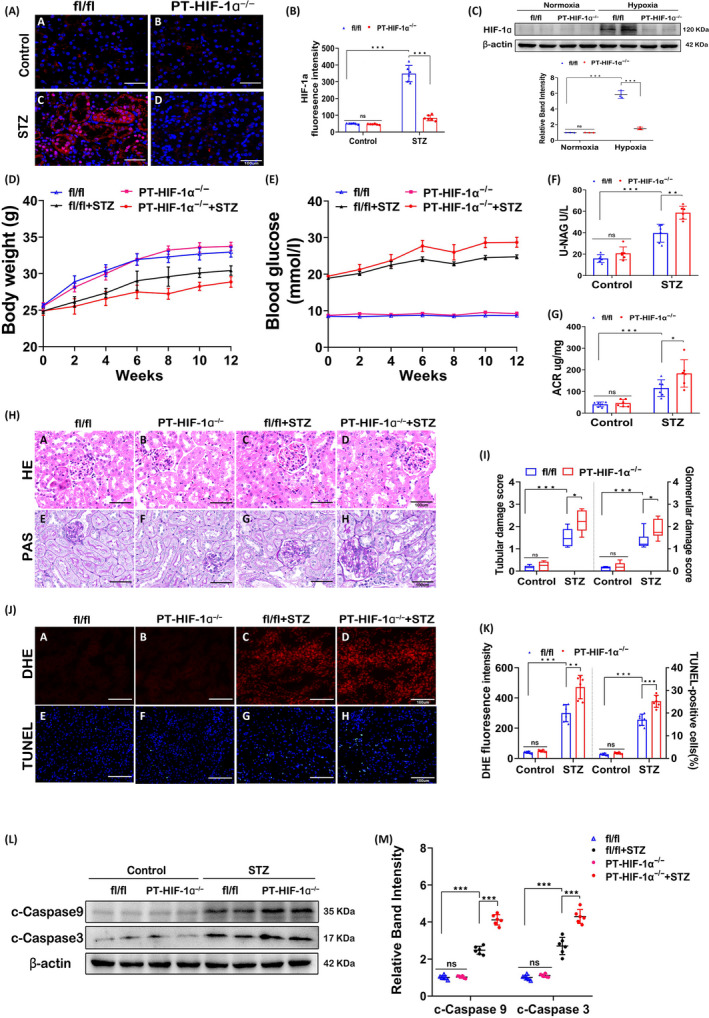
Conditional deletion of HIF‐1α in the proximal tubular epithelium worsened kidney injury. A and B, IF images of tissue from fl/fl mice, PT‐ HIF‐1α^−/−^ mice, diabetic fl/fl mice and diabetic PT‐ HIF‐1α^−/−^ mice. Red fluorescence indicates the expression of HIF‐1α in proximal tubular cells (A). Quantification analysis of fluorescence staining is shown (B). C, Primary proximal tubular epithelial cells were isolated from fl/fl mice and PT‐HIF‐1α^−/−^mice in the presence or absence of hypoxia. Expression of HIF‐1α was detected by Western blotting and quantification analysis of the related band intensity. n = 3. D‐G, Body weights (D), serum glucose levels (E), urinary ACR levels (F) and urinary NAG levels (G). H, Pathological changes in kidneys were demonstrated by HE (upper) and PAS (below) staining. I, The graph shows semiquantitative tubular and glomerular injury scores. J and K, DHE (upper) and TUNEL (below) assays (J) of kidney tissues and related semiquantitative results (K) are shown. L and M, Western blot (L) and quantification analysis (M) of c‐caspase‐9 and c‐caspase‐3 expression. ***P < .001; **P < .005; *P < .05. n = 6

IF staining showed a visible increase in the HIF‐1α expression in the diabetic groups, and in these groups, HIF‐1α expression was significantly decreased in PT‐HIF‐1α^−/−^ mice compared to fl/fl mice (Figure 1A,B). Western blot analysis reconfirmed the change in HIF‐1α expression in primary proximal tubular epithelial cells isolated from fl/fl mice and PT‐HIF‐1α^−/−^mice in the presence or absence of hypoxia (Figure 1C). The body weights of diabetic fl/fl mice and diabetic PT‐HIF‐1α^−/−^ mice were lower than those of non‐diabetic fl/fl mice (Figure 1D), and the opposite pattern was observed for the blood glucose level (Figure 1E). A significant increase in the urinary NAG and ACR was observed in diabetic mice compared to fl/fl mice, and diabetic PT‐HIF‐1α^−/−^ mice displayed the highest levels (Figure 1F,G). HE and PAS staining showed that kidney injury was further aggravated in diabetic PT‐HIF‐1α^−/−^ mice when comparing with diabetic fl/fl mice (Figure 1H). As shown in Figure 1I, the scores of evaluation of tubular and glomerular damage in diabetic PT‐HIF‐1α^−/−^ mice were the highest. Moreover, the DHE probe and TUNEL assay revealed notable increases in ROS production and apoptosis, respectively, in diabetic PT‐HIF‐1α^−/−^ mice compared to diabetic fl/fl mice (Figure 1J,K). In addition, we found greatly increased expression of cleaved caspase‐9 and caspase‐3 in diabetic PT‐HIF‐1α^−/−^ mice compared with diabetic fl/fl mice (Figure 1L,M).

### 3.2 **Mitochondrial fission is exacerbated in the tubular cells of diabetic PT‐HIF‐1α** ^−/−^ **mice**

**Figure 2:**
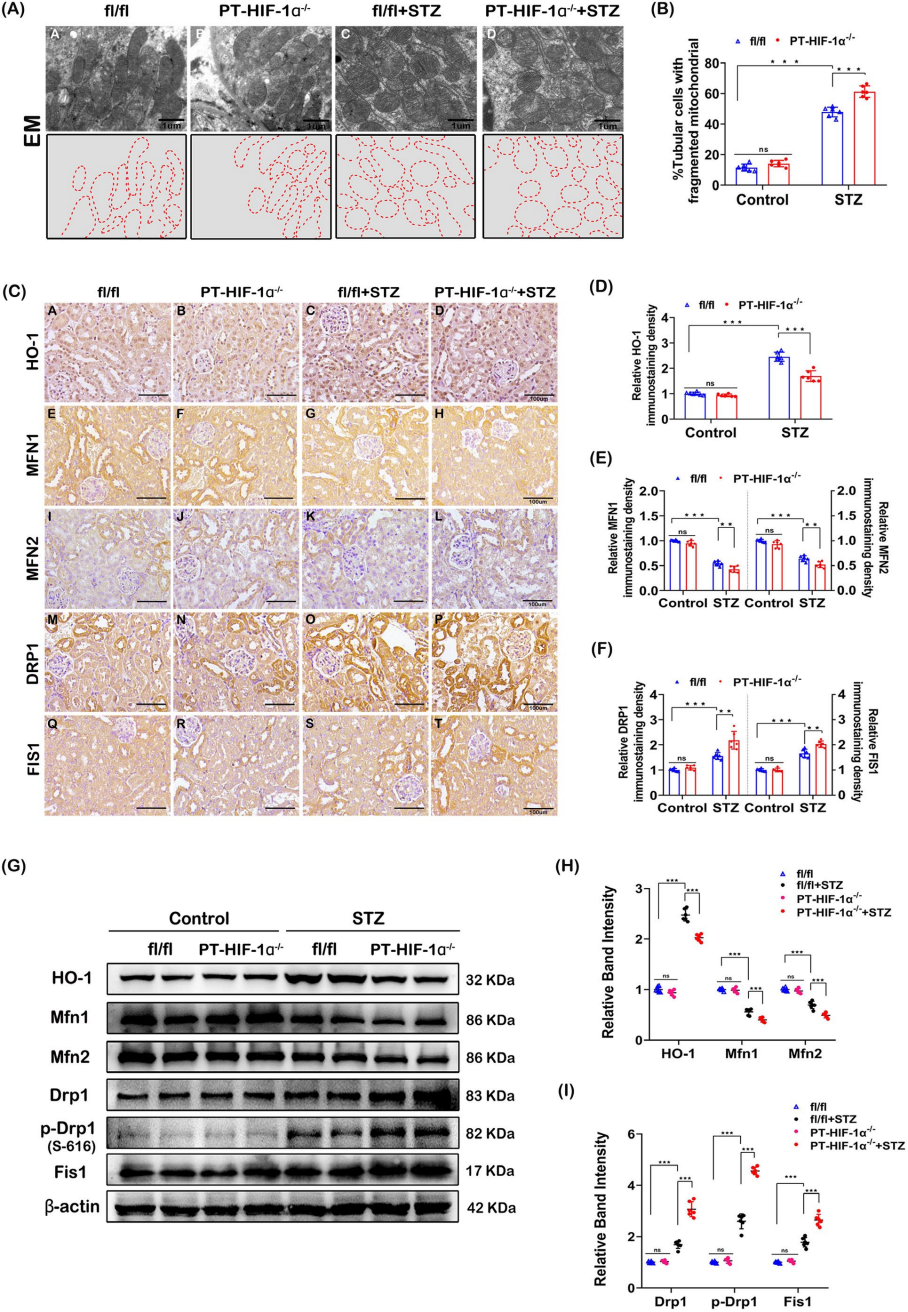
Alterations in mitochondrial dynamics in diabetic PT‐ HIF‐1α^−/−^ mice. A, EM micrographs showing fragmented mitochondria in renal tubules (upper). The aforementioned EM micrographs of mitochondria were traced (below). B, Histogram showing the percentage of fragmented mitochondria. C, IHC staining of kidney tissue revealed the expression of HO‐1, Mfn1, Mfn2, Drp1 and Fis1. D‐F, Semiquantification of the protein levels from the IHC shown staining in C. G‐I, Representative Western blot bands (G) and relative band densities (H and I) indicating HO‐1, Mfn1, Mfn2, Drp1, p‐Drp1(S‐616) and Fis1 expression. ***P < .001; **P < .005; *P < .05. n = 6

Mitochondrial fragmentation was increased in diabetic mice compared with fl/fl mice, while it was remarkably elevated in diabetic PT‐HIF‐1α^−/−^ mice (Figure 2A,B). IHC staining showed that HO‐1 expression was substantially increased in diabetic mice compared to non‐diabetic mice and was clearly reduced in diabetic PT‐HIF‐1α^−/−^ mice compared to diabetic fl/fl mice (Figure 2C, D). Furthermore, the expression levels of mitochondrial profusion proteins Mfn1 and Mfn2 were significantly declined in diabetic PT‐HIF‐1α^−/−^ mice compared to diabetic fl/fl mice (Figure 2C, E). In contrast, in the expression of mitochondrial profission proteins expression, like Drp1, p‐Drp1 and Fis1, were increased in diabetic PT‐HIF‐1α^−/−^ mice compared to diabetic fl/fl mice (Figure 2C, F). The above changes were reconfirmed by Western blot and related quantitative analysis (Figure 2G‐I).

### 3.3 Hypoxia induces the expression of HIF‐1α and mitochondrial fission and enhances mitochondrial membrane potential loss, ROS generation and apoptosis in HK‐2 cells

**Figure 3:**
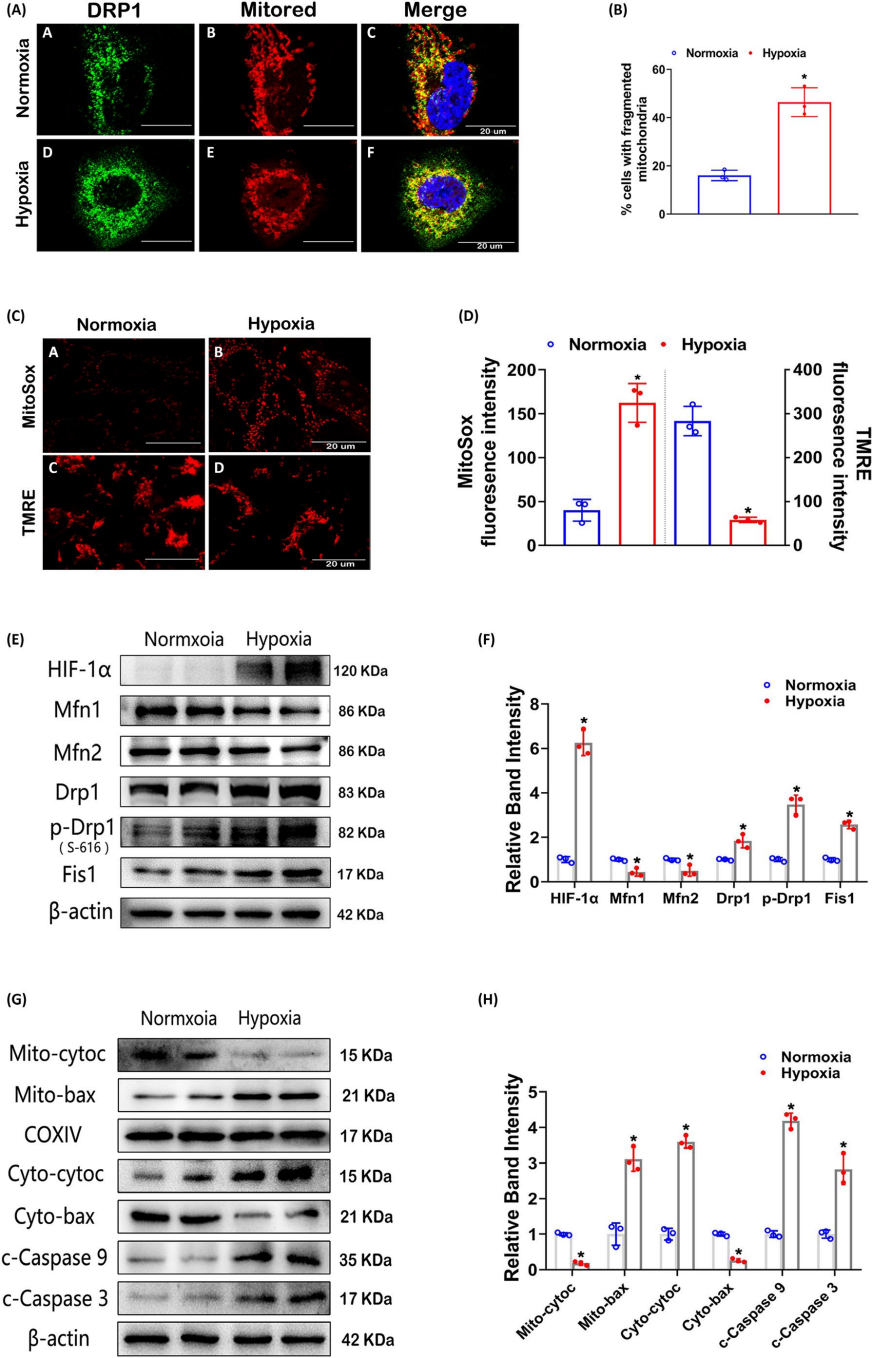
Mitochondrial morphological changes, apoptosis and related protein expression in HK‐2 cells in hypoxia. A, In HK‐2 cells treated with or without CoCl_2_, IF staining was performed with an anti‐Drp1 antibody and MitoTracker Red. The yellow dots represent the colocalization of Drp1 and mitochondria. B, Quantitative analysis of mitochondrial fragmentation was performed. C, Confocal images of MitoSox Red and TMRE staining indicating mitochondrial ROS generation and the mitochondrial membrane potential (△Ψm), respectively. D, Quantitative analyses of MitoSox Red and TMRE staining. E, Western blot analysis of HIF‐1α, Mfn1, Mfn2, Drp1, p‐Drp1(S‐616) and Fis1 expression in HK‐2 cells in normoxia or hypoxia. (F) Bar graph showing the quantitative analysis results of the relative band densities. G and H, Representative Western blot bands (G) and quantitative analysis (H) of mito‐Cyt.C, mito‐Bax, cyto‐Cyt.C, cyto‐Bax, c‐caspase‐9 and c‐caspase‐3. *P < .05 vs the normoxia group. n = 3

CoCl_2_ was applied to HK‐2 cells to mimic hypoxic conditions^28^; this method has previously been used to establish a cell model of DN.^30^ Mitochondria exhibited filamentous shapes in HK‐2 cells in normoxia, whereas marginally elevated mitochondrial fragmentation with short rods morphology was observed in hypoxia. These alterations were accompanied by increasing translocation of Drp1 to mitochondria, and yellow fluorescence revealed colocalization of Drp1 and mitochondria (Figure 3A,B). Moreover, mitochondrial ROS generation was remarkably increased (Figure 3C (upper) and D (left)) and the mitochondrial membrane potential was obviously decreased (Figure 3C (below) and D (right)) in hypoxic HK‐2 cells. Compared to normoxia, HK‐2 cells in hypoxia displayed high expression of HIF‐1α, and the expression of Mfn1 and Mfn2 was decreased; however, the levels of Drp1, p‐Drp1 and Fis1 were elevated in hypoxia (Figure 3E,F). In addition, Western blot analysis demonstrated mitochondrial cytochrome C level was reduced in hypoxia, while the level of mitochondrial Bax was notably increased. In contrast, among cytoplasmic proteins, hypoxia induced upregulation of Cytochrome C and downregulation of Bax. The correlative changes explained the increased translocation of mitochondrial cytochrome C to the cytoplasm and cytoplasmic Bax to mitochondria, which led to apoptosis. The extraction efficiency of mitochondrial and cytoplasmic proteins was shown in Figure S1. Moreover, hypoxia induced pro‐apoptotic proteins expression, such as cleaved caspase‐9 and caspase‐3 (Figure 3G,H).

### 3.4 Disruption of HIF‐1α exacerbates hypoxia‐induced mitochondrial damage in HK‐2 cells

**Figure 4:**
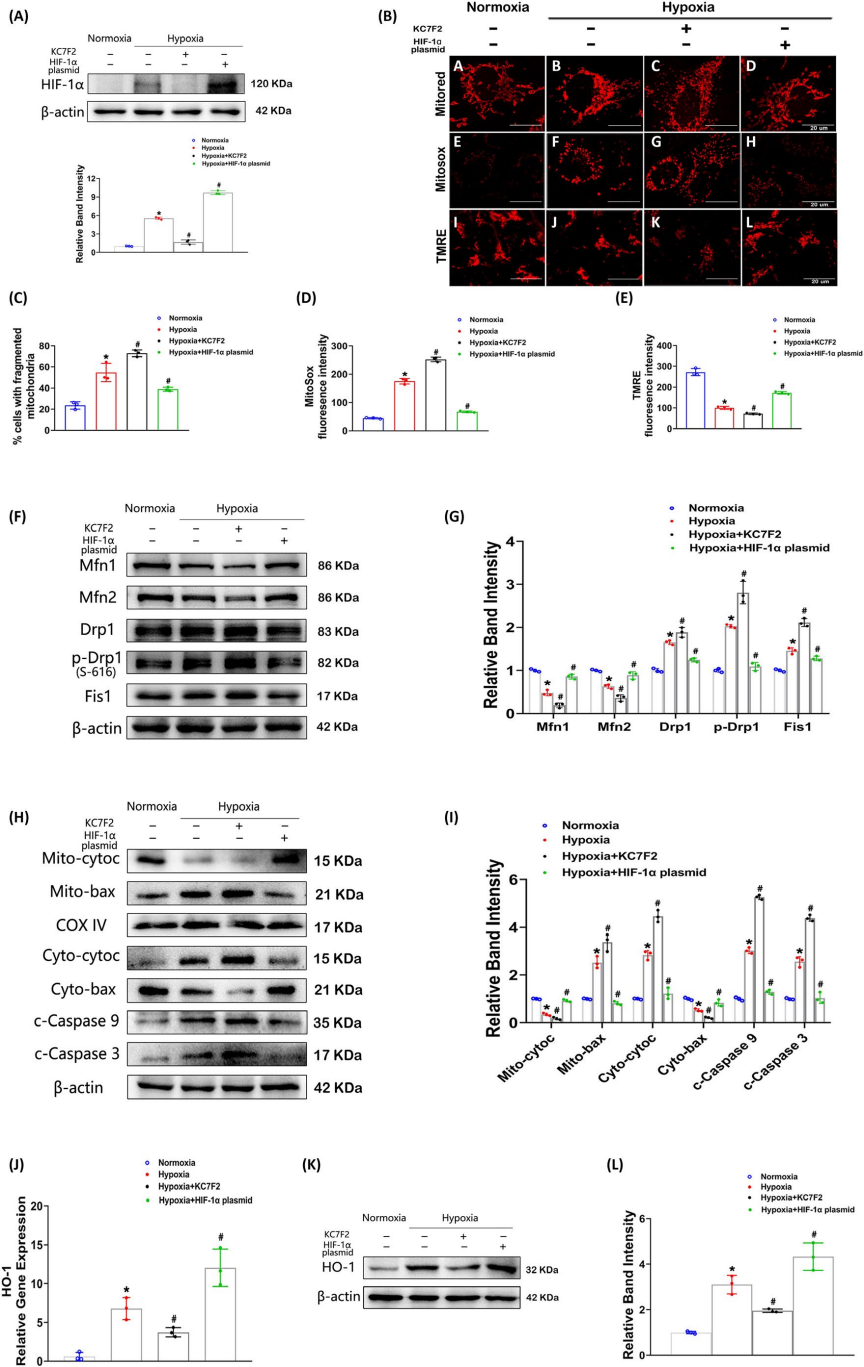
Effects of HIF‐1α on hypoxia‐induced mitochondrial damage in HK‐2 cells. A, Representative Western blot bands indicating HIF‐1α expression in HK‐2 cells treated with KC7F2 (a HIF‐1α inhibitor) or transfected with a HIF‐1α plasmid in hypoxia. B, Representative mitochondrial morphology, ROS generation and mitochondrial membrane potential as detected by MitoTracker Red (upper), MitoSox Red (middle) and TMRE (below) staining, respectively. C‐E, Histograms depicting mitochondrial fragmentation (C), the ROS production (D) and mitochondrial membrane potential (E). F and G, Representative Western blot bands (F) and relative densities (G) of Mfn1, Mfn2, Drp1, p‐Drp1(S‐616) and Fis1 expression in HK‐2 cells treated as described in A. H and I, Expression of mito‐Cyto.C, mito‐Bax, cyto‐Cyto.C, cyto‐Bax, c‐caspase‐9 and c‐caspase‐3 was revealed by Western blotting(H) and related densitometric analysis (I). J, The HO‐1 mRNA levels in HK‐2 cells were measured by real‐time PCR analysis. K and L, The protein expression of HO‐1 was assessed by Western blotting (K) and quantitative analysis (L). *P < .05 vs the normoxia group; ^#^ *P* < .05 vs the hypoxia group. n = 3

To explore the effects of HIF‐1α, HK‐2 cells were cultured in hypoxia and treated with the HIF‐1α inhibitor KC7F2 or transfected with a HIF‐1α plasmid. As shown in Figure 4A, KC7F2 treatment reduced hypoxia‐induced HIF‐1α expression, while transfection with the HIF‐1α plasmid elicited marked overexpression of HIF‐1α in cells. Moreover, KC7F2 treatment further exacerbated mitochondrial fragmentation, while overexpression of HIF‐1α restored mitochondrial morphology compared to that in hypoxia (Figure 4B (upper) and C). The same trends in mitochondrial ROS generation were found by MitoSox Red staining (Figure 4B (middle) and D), while the opposite trends in the mitochondrial membrane potential were assessed by TMRE staining (Figure 4B (below) and E). In addition, KC7F2 blocked the activity of mitochondrial complex I, which further indicated disruption of HIF‐1α exacerbated hypoxia‐induced mitochondrial damage (Figure S2A). Additionally, the levels of Mfn1 and Mfn2 were lower but those of Drp1, p‐Drp1 and Fis1 were higher in KC7F2‐treated cells than in hypoxic cells. However, overexpression of HIF‐1α reversed these changes (Figure 4F,G). Furthermore, cells cultured with KC7F2 under hypoxia further accelerated hypoxia‐induced released of mitochondrial Cytochrome C and translocation of cytoplasmic Bax. As expected, HIF‐1α overexpression reversed the consequences of hypoxia (Figure 4H,I). These treatments had similar effectiveness on cleaved caspase‐9 and caspase‐3 expression (Figure 4H,I). As shown by the real‐time PCR data in Figure 4J, a remarkably decrease in HO‐1 mRNA expression was displayed in KC7F2‐treated cells, whereas overexpression of HIF‐1α further increased the HO‐1 mRNA expression compared to that in hypoxia. Moreover, Western blot analysis revealed similar protein expression patterns (Figure 4K,L), indicating that HIF‐1α regulates HO‐1 expression directly.

### 3.5 Lack of HIF‐1α aggravates mitochondrial dysfunction in HK‐2 cells exposed to hypoxia through HO‐1

**Figure 5:**
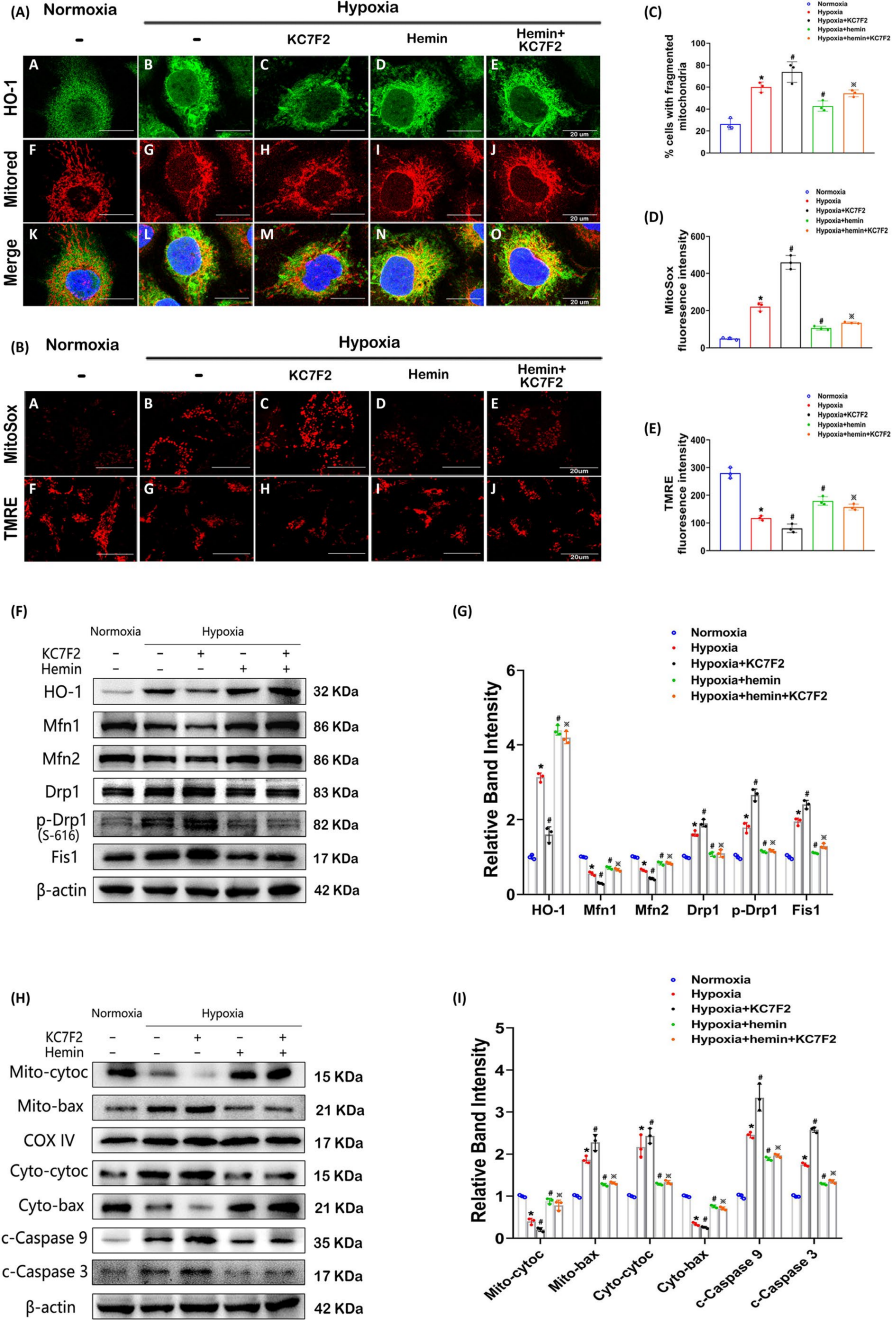
Effects of HO‐1 on HIF‐1α–regulated mitochondrial fragmentation, ROS generation and apoptosis in HK‐2 cells. A, Confocal images showing HO‐1 expression (upper) and mitochondria (middle) in HK‐2 cells in the normoxia, hypoxia, hypoxia + KC7F2, hypoxia + hemin and hypoxia + hemin+KC7F2 groups. Colocalization of HO‐1 with mitochondria was assessed (bellow). B, Confocal images of HK‐2 cells stained with MitoSox Red and TMRE. C‐E, The proportion of fragmented mitochondria (C) and the fluorescence intensity of MitoSox Red (D) and TMRE (E) were quantified and are shown in the histogram. F and G, Western blot analysis showing the protein expression levels of HO‐1, Mfn1, Mfn2, Drp1, p‐Drp1(S‐616) and Fis1 in HK‐2 cells treated as indicated (F), and the bands were analysed by densitometry(G). H and I, Western blot analysis of the levels of Bax, Cyt.C, c‐caspase‐9 and c‐caspase‐3 (H) and densitometric analysis of the bands (I). *P < .05 vs the normoxia group; ^#^ *P* < .05 vs the hypoxia group; ^※^ *P* < .05 vs the KC7F2 treatment group. n = 3

Cellular IF staining showed that HO‐1 agonist hemin further increased HO‐1 expression compared to that in hypoxia and that KC7F2 treatment could not block the effect of hemin (Figure 5A). Compared to hypoxia, hemin intervention restored mitochondrial morphology, and cotreatment with hemin and KC7F2 abolished the mitochondrial fission induced by KC7F2 (Figure 5A (middle) and C). Importantly, the changes observed by double‐staining of HO‐1 and MitoTracker Red were confirmed by confocal microscopy (Figure 5A (below)), revealing that HO‐1 may target mitochondria and impact them. The changes in mitochondrial ROS production were consistent with the mitochondrial morphology variations (Figure 5B (upper) and D), but the changes in the mitochondrial membrane potential and ATP content exhibited the opposite pattern (Figure 5B (below), E and Figure S2B). Western blot analysis reconfirmed the changes in HO‐1 expression, which were similar to those indicated by IF staining. Hemin increased the expression of Mfn1 and Mfn2 but inhibited that of Drp1, p‐Drp1 and Fis1. The negative impacts of KC7F2 were reversed by cotreatment with KC7F2 and hemin (Figure 5F,G). Moreover, the release of mitochondrial cytochrome C, the translocation of cytoplasmic Bax and the expression of cleaved caspase‐9 and caspase‐3 expression induced by hypoxia exposure were restored by hemin treatment. Furthermore, cotreatment with KC7F2 and hemin reversed the effects of KC7F2 treatment (Figure 5H,I).

## 4 DISCUSSION

**Figure 6:**
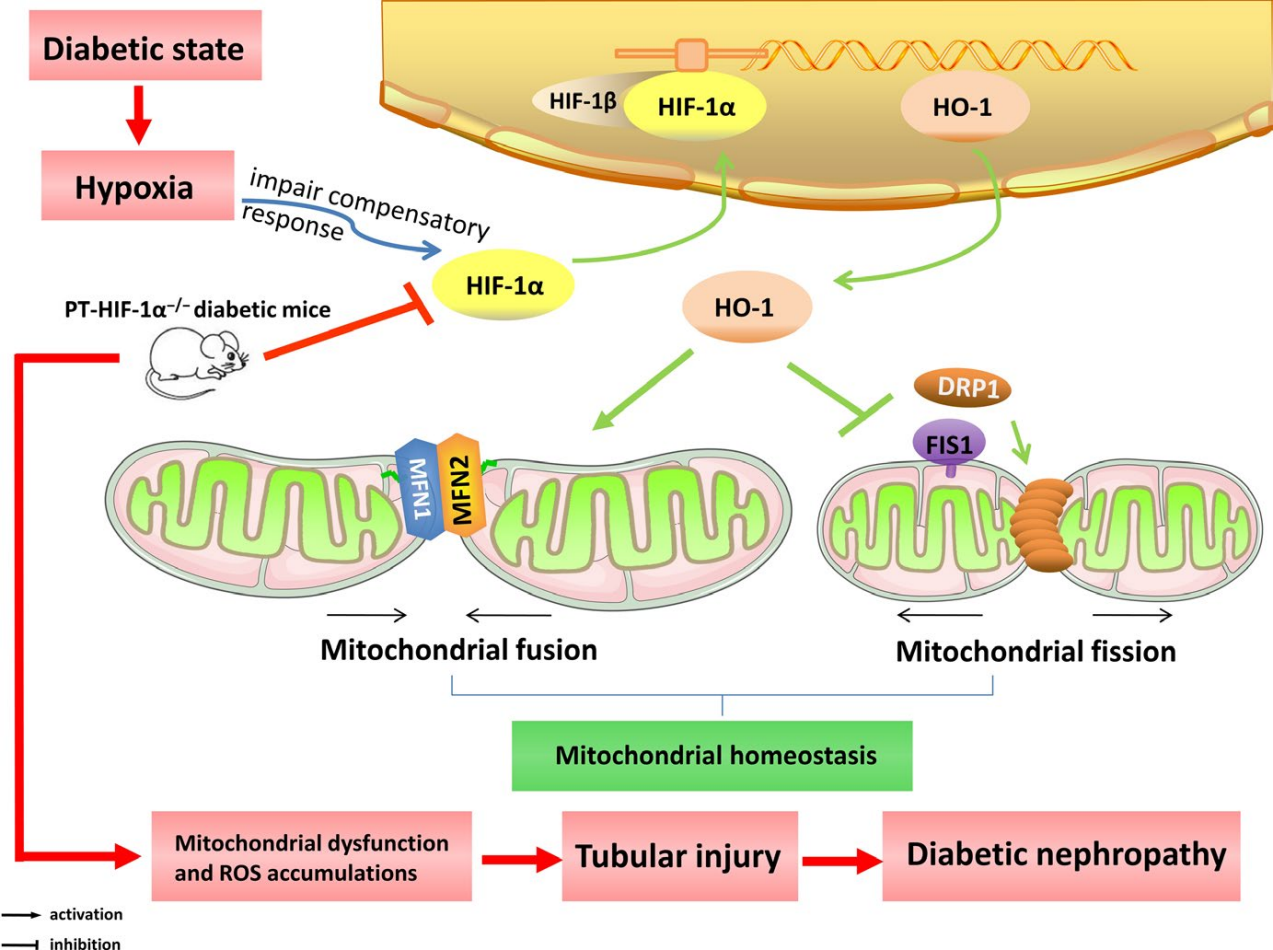
HIF‐1α is a transcription factor that regulates HO‐1 gene expression, and HO‐1 facilitates mitochondrial fusion (Mfn1 and Mfn2) and inhibits mitochondrial fission (Drp1 and Fis1), thus maintaining mitochondrial homeostasis. Under diabetic conditions, kidney tissues are hypoxic, which induces the expression of HIF‐1α through an impaired compensatory response. However, tubular‐specific deletion of HIF‐1α in the kidneys of diabetic mice exacerbated mitochondrial dysfunction and ROS accumulation and then caused tubular damage, thereby leading to diabetic kidney injury

This study demonstrated that HIF‐1α improved mitochondrial dysfunction and restricted mitochondria‐dependent apoptosis in tubular cells of DN via the HO‐1 pathway (Figure 6). This study provided the first demonstration of the protective role of HIF‐1α in tubular cell injury in mice with STZ‐induced DN. Moreover, a novel mechanism was proposed wherein the HIF‐1α/HO‐1 pathway is the pivotal pathway mediating tubular cell mitochondrial dynamics in DN.

Many studies have demonstrated that hypoxia occurs when there is an imbalance between oxygen supply and consumption, and this imbalance is deemed the major driver of DN.^1^, ^3^ In view of this information, we attempted to define the relationship between hypoxia and kidney damage in DN. Semenza et al^7^ were the first to report on HIF‐1, and their study expanded on the identify of HIF‐1 as a heterodimer that can respond to hypoxia. The investigation of HIF‐1α is particularly valuable in kidney disease since HIF‐1α is expressed predominantly in tubular cells, and tubules are susceptible to hypoxia.^8^, ^31^, ^32^ Furthermore, studies have shown that HIF‐1α governs the initial adaptive response to hypoxia.^3^, ^8^ Moreover, recent observations have shown that proximal tubular cells are the initiators of and critical therapeutic targets in diabetic kidney disease.^32^, ^33^, ^34^, ^35^ To explore the effects of HIF‐1α on tubular damage in DN, mice with proximal tubular cell‐specific HIF‐1α ablation were generated, and these mice were treated with STZ to establish a mouse model of DN. The absence of HIF‐1α in proximal tubular cells aggravated the kidney damage as evidenced by alterations in morphology and function. These data revealed that HIF‐1α plays a protective role in diabetic kidneys, consistent with the observation that HIF‐1α deficiency promotes renal injury in DN.^36^, ^37^

Interestingly, studies have suggested that HIF‐1α can influence mitochondrial morphology and function.^12^, ^14^, ^15^ In addition, our previous studies proved that mitochondria play a vital role in DN, especially regarding mitochondrial dynamics.^16^, ^17^, ^29^, ^38^ However, whether mitochondria are involved in the protective effect of HIF‐1α against tubular injury in DN is still unknown. To explore this possibility, CoCl_2_ was applied to HK‐2 cells to mimic hypoxia because it is widely used and recognized as a cell model for chemically induced hypoxia; this model is relatively simple, and the conditions are easy to control. Moreover, treatment with an appropriate concentration of CoCl_2_ simulates a hypoxic state, including the induction of HIF‐1α expression^39^ in cells such as tubular cells.^28^ In the present study, we found that the lack of HIF‐1α in tubular cells of diabetic kidneys aggravated mitochondrial injury.

This study showed that the expression of HIF‐1α was increased in DN kidneys of DN, consistent with the results of other studies.^3^, ^9^ A remaining question is why the level of HIF‐1α is elevated in diabetic kidneys when it has protective impacts in renal tissue. The increasing expression and activity of HIF‐1α is one type of impaired compensatory response.^40^ After the level of HIF‐1α is increased, its downstream target genes are activated to suppress hypoxia‐induced damage to cells and organs, which exerts renoprotective effects.^9^, ^21^ In addition, evidence from recent studies indicates that the activity of HIF in tubules is a lack of compensatory response in DN.^9^, ^30^ The possible reasons are as follows: first, superoxide (O_2_^−^) may reduce HIF‐1 activity by inducing α subunit degradation^3^, ^40^, ^41^; second, hyperglycaemia could suppress HIF‐1α responsive transactivation in tubular cells.^42^, ^43^, ^44^ In vitro experiments confirmed that the high glucose‐stimulated increase in HIF‐1α expression was not significant in tubular cells.

Mitochondrial dynamics describes the two features of mitochondria, which manifest as fusion and fission.^45^, ^46^, ^47^ In addition, HO‐1 may be the intermediary molecule linking HIF‐1α and mitochondrial dynamics. HO‐1 is a target gene of HIF‐1α^22^ and exerts protective effects against kidney damage under diabetic conditions.^23^ Studies have shown that HO‐1 exerts anti‐apoptotic, antioxidant, anti‐nitrosative and anti‐inflammatory effects,^48^, ^49^, ^50^ by reducing the level of the pro‐apoptotic protein Bax and proinflammatory/prooxidant protein iNOS,^49^, ^50^, ^51^ and increasing the level of the anti‐apoptotic protein Bcl‐xl and anti‐nitrosative protein bilirubin.^49^, ^50^, ^52^ Importantly, recent studies have verified that HO‐1 activity affects the function and morphology of mitochondria, especially mitochondrial dynamics.^53^, ^54^, ^55^ On the basis of these findings, we sought to determine whether HO‐1 is the key modulator of HIF‐1α‐mediated regulation of mitochondrial dynamics in tubular cells. As expected, under hypoxic conditions, HK‐2 cells exhibited increased mitochondrial fission, mitochondrial ROS generation and apoptosis, while these effects were further enhanced by HIF‐1α inhibitor treatment. While clearly of great importance, in HK‐2 cells cotreated with a HIF‐1α inhibitor and a HO‐1 agonist after exposure to hypoxia the HO‐1 agonist rescued the cells from the negative impact of the HIF‐1α inhibitor. These results suggest that HIF‐1α is the pivotal node upstream of HO‐1 expression, identifying a novel pathway through which HIF‐1α modulates mitochondrial dynamics via HO‐1 in tubular cell injury in DN.

A recent question addresses the specific molecular mechanism by which HO‐1 modulates mitochondrial dynamics. Studies speculated that HO‐1 can regulate mitochondrial dynamics^53^, ^54^, ^56^ and showed that HO‐1/CO may mediate mitochondrial dynamics in leukaemia.^55^ These results indicate that HO‐1‐mediated regulation of alterations in mitochondrial dynamics in tubular cells of DN may occur through the HO‐1/CO pathway, a possibility that requires further investigation. Iron overload can damage mitochondria, and HO‐1 participates in iron metabolism by degrading haem into ferrous iron.^57^, ^58^ However, the increase in HO‐1 is also accompanied by ferritin upregulation and leads to effluxion of cellular iron.^59^, ^60^ Most importantly, the increase in HO‐1 causes a decrease in intracellular free iron content and thus mitigates mitochondrial dysfunction.

This study provides a novel perspective on HIF‐1α in DN‐by not only developing a new research field in diabetic kidney disease but also laying a foundation for the identification of promising therapeutic targets. In summary, HIF‐1α plays a vital role in tubular cell injury in DN and is thus a potential therapeutic target.

## CONFLICT OF INTEREST

No competing interests related to this article are reported.

## AUTHOR CONTRIBUTIONS

NJ performed the experiments, generated the data and wrote the manuscript. HZ, YCH, SX, LFZ and XFX performed statistical analyses of the data and discussed the results of the manuscript. LL, MY, YX, LW and PG partially edited the manuscript. YL provided technical support for this study. LS is the guarantor of this study, who conceived and designed this study and edited and discussed this manuscript.

## Supporting information

Fig S1

Fig S2

Method S1

## Data Availability

The data that support the findings of this study are available from the corresponding author upon reasonable request.
