# Supplementary material for: HIF‐1α ameliorates tubular injury in diabetic nephropathy via HO‐1–mediated control of mitochondrial dynamics
Source: Cell Prolif. 2020 Sep 25;53(11):e12909. doi: 10.1111/cpr.12909 (PMC7653251; doi:10.1111/cpr.12909)
Supplement: Supplementary file 3 — Method S1 [file CPR-53-e12909-s003.docx]

**Supplementary Information**

**Methods**

**1.1 Isolation of PTECs**

Primary PTECs were isolated from kidney tissue as described previously. Briefly, to clean the kidneys (such as blood cells), sterile saline were used to flush the tissue in vivo. The optimum volume of the cortices tissue was 1mm3. Cut the cortices into pieces and wash them for three times with sterile saline. To digest the tissue pellet, Hank’s Balanced Salt Solution (HBSS) and collagenase II (2mg/ml) were mixed and used for 50 min. Then 100 um and 40um sieves were used to filtrate the digested cortical tissue and the filtered solution was centrifuged at 1000 rpm for 5 min.K1 medium(19) was used to resuspend the cell pellet, containing 5% fetal bovine serum(FBS). Collagen-coated Petri dishes (Corning® BioCoat^TM^, USA) were used to incubate the cell suspension in a cell incubator.

**1.2 Detection of mitochondrial complex I activity**

Complex I Enzyme Activity Microplate Assay Kit (ab109721) was used to determine mitochondrial complex I activity according to kit instructions. Briefly, cells proteins were collected and the concentration was measured. And 1/10 volume of detergent to extract detergent on ice with 30 min, then collect the supernatant and used for detection in the preparation buffer. And the complex I activity increases absorbance at 450 nm.

**1.3 Quantification of ATP content**

The content of ATP in HK-2 cell was measured following the instructions of ATP assay kit from Beyotime (China). The cells were collected with prepared buffer and centrifuged at 12,000 ×g for 5 min at 4°C. Then extract supernatant and mix specified volume of the supernatant and luciferase reagent for ATP assay. The content of ATP was linearly related to the emitted light.

**Figure legends**

**Figure 1** The efficiency of mitochondrion and cytoplasm isolation of HK-2 cells. In HK-2 cells treated with or without CoCl_2_, western blot analysis of Bax、 Cyto.C、 COX IV、β-actin、GAPDH and PCNA expression in mitochondria or cytoplasm.

**Figure 2** Effects of HIF-1α on the activity of complex I and the production of ATP in HK-2 cells. (A) Quantification analysis of mitochondrial complex I activity in HK-2 cells treated with KC7F2 (a HIF-1α inhibitor) or transfected with a HIF-1α plasmid in hypoxia. (B) Quantification analysis showing the ATP content in HK-2 cells in normoxia, hypoxia, hypoxia+KC7F2, hypoxia+hemin, and hypoxia+hemin+KC7F2 groups.
